# The Relationship Between Family Caregivers’ Anticipatory Grief and Severity of Dementia.

**DOI:** 10.1093/geroni/igab046.3449

**Published:** 2021-12-17

**Authors:** Nicole Gavin, Mu Shan, Shelly Johns, Katherine Judge, Nicole Fowler

**Affiliations:** 1 Indiana University School of Medicine, IU Center for Aging Research, Indianapolis, Indiana, United States; 2 Indiana University, Indianapolis, Indiana, United States; 3 Indiana University School of Medicine, Center for Health Services Research, Indianapolis, Indiana, United States; 4 Cleveland State University, Cleveland, Ohio, United States

## Abstract

Anticipatory grief is the process of experiencing normal bereavement before the physical death of a significant person. To date, anticipatory grief has been related to higher levels of caregiver depression, anxiety, subjective burden, and poorer problem solving. Additionally, higher levels anticipatory grief are observed in caregivers of those with Alzheimer’s Disease and Related Dementias (ADRD) compared to caregivers of those with mild cognitive impairment, implying a relationship between disease severity and caregiver anticipatory grief. Analyses were performed on data for ADRD caregivers (n=56) enrolled in the IU Telephone Acceptance and Commitment Therapy for Caregiver (TACTICs) trial; an RCT evaluating an ACT intervention for ADRD caregivers with anxiety. Inclusion criteria included identifying as the primary caregiver of an ADRD patient, and clinically significant anxiety (GAD7 score >10). The average age of caregivers was 61.9 years, 41.1% were spouses, 83.9% were white and 14.3% were black. Mean anticipatory grief scores were notably higher (84.6) compared to the previously reported means across the literature (74.9). Using multiple regression models, we determined a caregivers’ anticipatory grief, as measured by the anticipatory grief scale, is not significantly associated with the patients’ dementia severity, as measured by the Dementia Severity Rating Scale (DSRS) (p=0.66), Results revealed that higher levels of caregiver burden, as measured by the Zarit Burden Index, are significantly associated with more anticipatory grief (p< 0.01). Understanding these relationships contributes to a better understanding of ADRD caregivers, identifying “high-risk” caregivers, and informing future interventions and care.

